# Incidence and Outcomes of Early Cancers After Kidney Transplantation

**DOI:** 10.3389/ti.2022.10024

**Published:** 2022-05-03

**Authors:** A. Krishnan, G. Wong, A. Teixeira-Pinto, W. H. Lim

**Affiliations:** ^1^ Department of Renal Medicine, Sir Charles Gairdner Hospital, Perth, WA, Australia; ^2^ Sydney School of Public Health, University of Sydney, Sydney, NSW, Australia; ^3^ Centre for Transplant and Renal Research, Westmead Hospital, Sydney, NSW, Australia; ^4^ Centre for Kidney Research, The Children’s Hospital at Westmead, Sydney, NSW, Australia; ^5^ School of Medicine, University of Western Australia, Perth, WA, Australia

**Keywords:** kidney transplantation, early cancer, cancer, ANZDATA, registry, cancer outcome, cancer death

## Abstract

Outcomes of early cancers after kidney transplantation are not well-understood. We included recipients of first live and deceased donor kidney transplants who developed *de novo* cancers in Australia and New Zealand between 1980–2016. We compared the frequency and stage of specific cancer types that developed early (≤12-months) and late (>12-months) post-transplantation. Risk factors for death were evaluated using multivariable Cox regression analyses. Of 2,759 recipients who developed *de novo* cancer, followed-up for 40,035 person-years, 243 (8.8%) patients were diagnosed with early cancer. Post-transplant lymphoproliferative disease, urinary cancers and melanoma were the most common cancer types (26%, 18%, and 12%) and the majority were either *in-situ* or locally invasive lesions (55%, 84%, and 86%). Tumors arising early from the gastrointestinal and respiratory systems were uncommon but aggressive, with 40% presenting with metastatic disease at time of diagnosis. Overall, 32% of patients with early cancers died within a median of 4.7 months (IQR:0.6–16) post-diagnosis and 91% were cancer-related deaths. Older recipient and donor age were associated with an increased risk of all-cause death. Early cancers, though infrequent in kidney transplant recipients, are associated with poor outcomes, as nearly 1 in 3 died from cancer-related death; with majority of deaths occurring within 12-months of cancer diagnosis.

## Graphical Abstract

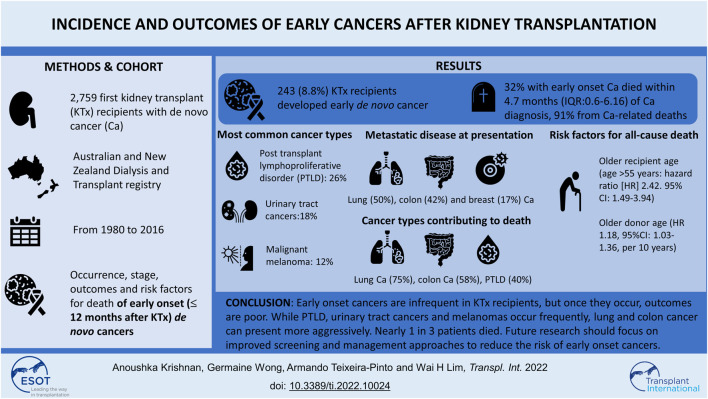

## Introduction

Cancer is a leading cause of death for many patients after kidney transplantation (1, 2). Compared to age and sex matched general population, cancer incidence and mortality rates are 2-3 times higher among transplant recipients (3). Epidemiological data have reported the mean time from transplantation to cancer diagnoses is approximately 6 years, suggesting that intensity of immunosuppression and cumulative drug exposure play key roles in cancer development (4, 5). However for some early cancers, such as post-transplant lymphoproliferative disease (PTLD) that commonly occur within a short timeframe after transplantation, the mechanistic pathways for cancer development may be different to those that occur later (5). Patients on dialysis are also at risk of certain cancers such as urinary tract cancer (6). Clinical practice guidelines recommend age-specific screening for potential transplant candidates and some guidelines suggest additional screening for kidney cancers in patients on dialysis (7). However, the sensitivity of these screening tests is imperfect (8) and may therefore, miss occult malignancies. Under the influence of immunosuppression, occult cancers may grow rapidly through deficiencies in tumor surveillance, and manifest early after transplantation.

Prior research has not quantified the burden and outcomes of these early cancers after transplantation. Knowledge of the epidemiology of these cancers and their risk factors for adverse outcomes will help to identify complex and high-risk patients and facilitate appropriate interventions such as targeted cancer screening in this at-risk population. In this study we aimed to compare the frequency, types, sites and stage of cancers that occurred early compared to those that occurred later after transplantation. We also compared the risk of cancer-related and all-cause death between recipients with early and late cancers and defined the risk factors for deaths in patients with early cancer.

## Materials and Methods

### Study Population

**FIGURE 1:**
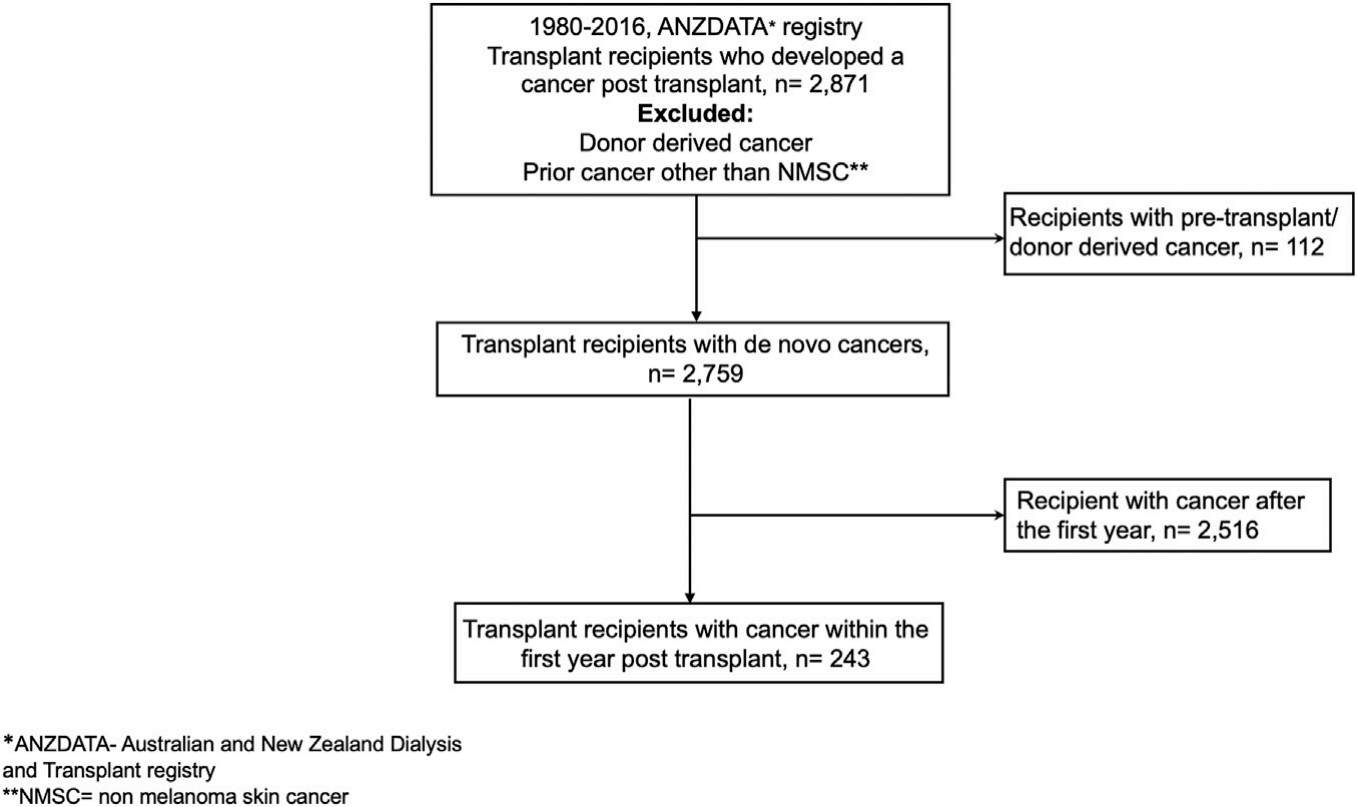
Participant flow.

Using data from the Australia and New Zealand Dialysis and Transplant (ANZDATA) registry, kidney failure patients who have received a first deceased and living donor kidney transplant in Australia and New Zealand between 1980 and 2016 and had developed “*de novo*” cancer after transplantation were included in the analyses. Recipients with a prior history of cancer (other than a history of non-melanoma skin cancer- NMSC) and known “donor-transmitted” and “donor-derived” cancers were excluded (Figure 1). *De novo* cancer was defined as a cancer that occurred in a kidney transplant recipient with no prior history of cancer before transplantation and included all cancer types except NMSC. Donor-transmitted cancers are those cancers which are present in the donated organ and tissue at transplantation, whereas donor-derived cancers are those that are of donor origin but developed *de novo* in the allograft after transplantation. Details of both donor-derived and donor-transmitted cancers are provided to the ANZDATA registry by the individual units. However, the ANZDATA registry does not verify whether the cancer cells were of donor origin.

The clinical and research activities being reported are consistent with the Principles of the Declaration of Istanbul as outlined in the “Declaration of Istanbul on Organ Trafficking and Transplant Tourism.” Ethics approval was obtained from the Human Research Ethics Committee of Western Australia, Australia. Written informed consents were sought from patients with kidney failure at time of entry into the registry, including the utilization of aggregate data for future research.

### Exposure

Recipients were categorized according to whether they had developed early cancer or late cancer post kidney transplant. Early-onset cancers were defined as those cancers occurring within the first 12-months post transplantation, whereas late-onset cancers were defined as those occurring 12-months after transplantation.

### Data Collection

Baseline characteristics recorded by the ANZDATA registry included donor factors of age, type and sex; recipient characteristics of age, sex, ethnicity, body mass index, waiting time prior to transplantation, comorbid conditions at time of transplantation (presence or absence of diabetes, coronary artery disease, cerebrovascular disease and peripheral vascular disease), primary causes of kidney failure; and transplant-related factors including the number of human leukocyte antigen (HLA) mismatches, total ischemic time (in hours), induction (none, interleukin-2 receptor therapy and T-cell depleting therapy) and initial immunosuppressive therapies (prednisolone, calcineurin-inhibitor and anti-metabolite therapies) and transplant era (categorized into 1980–1989, 1990–1999 and 2000–2016 transplant periods).

### Ascertainment of *De Novo* Cancers

*De novo* cancers occurring post-kidney transplantation were reported to the ANZDATA registry. The registry does not verify the histology of the *de novo* cancers, but the cancer records within the ANZDATA registry are accurate with a high concordance rate compared to those reported to the New South Wales Cancer Registry (9), a mandatory requirement for cancer reporting in New South Wales. *De novo* cancers are recorded according to cancer sites and cell types according to the International Classification of Disease for Oncology, edition 3, first revision (ICD-O-3.1) (10).

### Clinical Outcomes

The primary outcomes included the frequency, types, sites, stage (including presence of lymph node involvement and distant metastatic disease) and occurrence of cancer recurrence. Other outcomes included treatment of the *de novo* cancers in recipients with early cancers and comparison of the risk of cancer-related and all-cause death between recipients with early cancer and those with late cancer. We also defined the risk factors for all-cause deaths in recipients with early cancers.

### Statistical Analyses

Data were expressed as number (proportion), mean and standard deviation (SD) and median and interquartile range (IQR) where appropriate, with comparisons between groups by chi-square test, analysis of variance (ANOVA) and Kruskal–Wallis test, respectively. We compared the frequency, cancer types, stage and outcomes of patients who developed early cancers with those who developed cancers 12 months after transplantation. The treatment patterns, responses to treatment and outcome of early-onset cancers were also described. Kaplan Meier survival curves were constructed for all-cause and cancer-specific mortality in recipients with early cancers and stratified by site-specific cancer types. The log-rank test was used to test the trend of all-cause and cancer-specific survival functions across the cancer types. Survival time was censored at the date of the clinical outcome or on 31 December 2017. The cumulative survivals (and 95%CI) from the time of cancer diagnosis till the time of death were calculated for patients with early and late-onset cancers. Adjusted multivariable cox regression models were used to evaluate the risk factors for all-cause mortality in patients with early-onset cancers. Covariates with p-values of <0.25 in the unadjusted association for all-cause mortality were included in the multivariable analyses. Proportional hazard assumptions were checked, and two-way interactions were tested. The final model retained the covariates that remained significant after adjustment using a backward stepwise strategy. Variables included in the final multi-variable model included donor age, recipient age (stratified as <35, 35–55 and over 55 years), sex, race (Indigenous Australians, Maori and other), smoking status (stratified as current smoker, ex-smoker or non-smoker), induction immunosuppression, initial anti-metabolite therapy (none, azathioprine and mycophenolic acid) and transplant era.

Analyses were undertaken using SPSS V10 statistical software program (SPSS Inc., North Sydney, Australia), R (version 3.6) and STATA (version 11 StataCorp LP, College Station, TX). P-values of <0.05 were considered statistically significant.

## Results

### Study Population

Between 1980 and 2016, a total of 21,844 patients received a first kidney transplant. Of these, 2,871 kidney transplant recipients developed cancer(s) post-transplantation, with 2,759 (96.1%) recipients developing *de novo* cancers post-transplant and 112 (3.9%) with either pre-transplant or donor derived cancers, respectively. Of the 2,759 recipients with *de novo* cancers, 243 (8.8%) developed *de novo* cancers within the first 12 months post-transplantation (Figure 1). The median (IQR) patient-follow up time for all recipients was 13.4 years (7.84–20.44) resulting in 40,006 patient-years of follow up with shorter median (IQR) follow-up periods for those who developed early cancer (4.8 [1.6–10.9]) years with 1,699 patient-years of follow-up.

Baseline characteristics of the study population with early and late onset *de novo* cancers are shown in Table 1. Recipients who developed early cancer were older (mean [SD] age: 50.8 [15.4] vs. 45.3 [14.2] years, *p* < 0.001), more likely to have pre-transplant diabetes (16.5% vs. 10.5%, *p* = 0.002) and coronary artery disease (12.3% vs. 6%, *p* < 0.001) and received kidneys from older donors (mean [SD] age: 42.3 [17.1] vs. 35.7 [18.6] years, *p* < 0.001) compared to those who developed late cancers. Additionally, a higher proportion of early *de novo* cancers developed in later transplant era (after the year 2000) [63.8% vs. 33.8%, *p* < 0.001]. The incidence rate of early onset cancer was 0.01 (95%CI: 0.007, 0.013) per 1000-person-days between 1980–1989, 0.02 (95%CI: 0.013, 0.022) per 1000-person-days between 1990–1999 and 0.09 (95%CI: 0.08, 0.10) per 1000-person-days after the year 2000.

**TABLE 1:** Baseline characteristics of patients who developed early (within 12 months) and late (after 12 months) *de novo* cancer post-transplant (*n* = 2,759).

	Early cancers (*n* = 243, *n*, %)	Late cancers (*n* = 2,516, *n*, %)	p-values
Donor characteristics			
Age (years, mean [SD])	42.3 (17.1)	35.7 (18.6)	<0.001
Female gender (*n*, %)	118 (48.6)	1004 (39.9)	0.001
Type			0.03
Deceased	179 (73.7)	1927 (76.6)	
Live	64 (26.3)	589 (23.4)	
Recipient characteristics			
Age (years, mean [SD])	50.8 (15.4)	45.3 (14.2)	<0.001
Female gender (*n*, %)	98 (40.3)	1051 (41.8)	0.66
Race (*n*, %)			0.61
Caucasian	208 (85.5)	2224 (88.4)	
Aboriginals/Maori	11 (4.6)	87 (3.4)	
Others	24 (9.9)	205 (8.2)	
Diabetes (*n*, %)	40 (16.5)	265 (10.5)	0.002
Coronary artery disease (*n*, %)	30 (12.3)	152 (6.0)	<0.001
Peripheral vascular disease (*n*, %)	10 (4.1)	80 (3.2)	0.09
Cerebrovascular disease (*n*, %)	9 (3.7)	41 (1.6)	0.04
Smoker (*n*, %)			0.19
Non- smokers	116 (57.4)	1008 (52.4)	
Former smokers	67 (33.2)	655 (34.0)	
Current smokers	19 (9.4)	262 (13.6)	
Cause of ESKD (*n*, %)			0.03
Glomerulonephritis	104 (42.8)	1186 (47.1)	
Cystic	32 (13.2)	374 (14.9)	
Diabetes	30 (12.3)	201 (8.0)	
Vascular	16 (6.6)	96 (3.8)	
Analgesic nephropathy	11 (4.5)	107 (4.3)	
Others	50 (20.6)	552 (21.9)	
Viral serology			
CMV			<0.001
Negative	65 (26.7)	565 (22.5)	
Positive	138 (56.8)	1143 (45.4)	
Unknown	40 (16.5)	808 (32.2)	
EBV			<0.001
Negative	43 (17.7)	237 (9.4)	
Positive	134 (55.1)	1061 (42.1)	
Unknown	66 (27.2)	1218 (48.5)	
Immunology/transplant			
Waiting time (days, mean [SD])	868 (777)	774 (768)	0.07
Ischemic time (hours, mean [SD])	11.1 (7.4)	12.5 (7.9)	0.01
Transplant era (*n*, %)			<0.001
1980-1989	30 (12.3)	667 (26.5)	
1990-1999	58 (23.9)	998 (39.7)	
After 2000	155 (63.8)	851 (33.8)	
Induction immunosuppression			<0.001
None	131 (54)	1917 (76)	
Interleukin-2 receptor therapy	102 (42)	446 (18)	
T-cell depleting therapy	10 (4)	153 (6)	
Maintenance immunosuppression			
Steroids (Prednisolone)	226 (93)	2245 (89)	0.06
Calcineurin inhibitors			<0.001
None	16 (7)	297 (12)	
Cyclosporine	145 (60)	1833 (73))	
Tacrolimus	82 (33)	386 (15)	
Anti-metabolites			<0.001
None	27 (11)	321 (13)	
Azathioprine	60 (25)	1246 (49)	
Mycophenolate mofetil/sodium	156 (64)	949 (38)	

Recipients who developed early cancer in the latter era (after 2000) were older compared to those who developed cancer in the earlier eras (*p* ≤ 0.01, Supplementary Table S1). The proportion of incident kidney transplant recipients with early-onset cancers was similar across the three eras of 1980-1989 (0.8%), 1990–1999 (1.2%) and 2000–2016 (1.2%) (*p* = 0.36, Supplementary Figure S1).

### Cancer Types of Early-Onset and Late-Onset *De Novo* Cancers

The median (IQR) time to cancer onset was 205 days (107–298) in those with early-onset cancer compared to 2,083 days (1,675–4,914) in those with late-onset cancer. The three most common types of cancers in those who developed early *de novo* cancers were PTLD (25%), urinary tract cancers (18%) and malignant melanoma (12%). For late-onset cancers, the three most common types of *de novo* cancers were PTLD (14%), urinary tract cancers (13.6%) and melanomas (10.6%) (Table 2). Supplementary Table S2 demonstrates differences between recipients of living and deceased donor kidneys who developed early cancers. The distribution of the three most frequently occurring cancers (PTLD, urinary cancers and melanomas) were similar between the two groups.

**TABLE 2:** Distribution of cancer types amongst patients who developed early cancer compared to those who developed late cancer.

Cancer type (*n*, %)	Cancer within 12 months (*n* = 243)	Cancer after 12 months (*n* = 2,516)	p-value
Post-transplant lymphoproliferative disease	62 (25.5)	351 (14)	<0.001
Urinary tract cancer^a^	44 (18.1)	342 (13.6)	0.05
Melanoma	29 (11.9)	266 (10.6)	0.51
Other GI tract^b^	17 (7.0)	152 (6.0)	0.35
Genital^3^	15 (6.2)	262 (10.4)	0.04
Colorectal	12 (4.9)	197 (7.8)	0.10
Breast	12 (4.9)	143 (5.7)	0.23
Prostate	9 (3.7)	172 (6.8)	0.06
Lung	8 (3.3)	182 (7.2)	0.02
Thyroid	7 (2.9)	54 (2.1)	0.45
Brain	3 (1.2)	24 (1.0)	0.18
Lip	1 (0.4)	30 (1.2)	0.27
Unknown origin	2 (0.8)	70 (2.8)	0.006
Others	22 (9.1)	271 (10.8)	0.68

a Cancers of the kidney, ureters and bladder.

b Other gastrointestinal tract cancers (including gall bladder, small intestine, bile duct, pancreas, liver, stomach, esophagus).

c Cervix, ovaries, uterus, penile.

### Cancer Stage and Outcomes of Early-Onset and Late-Onset *De Novo* Cancers

**FIGURE 2:**
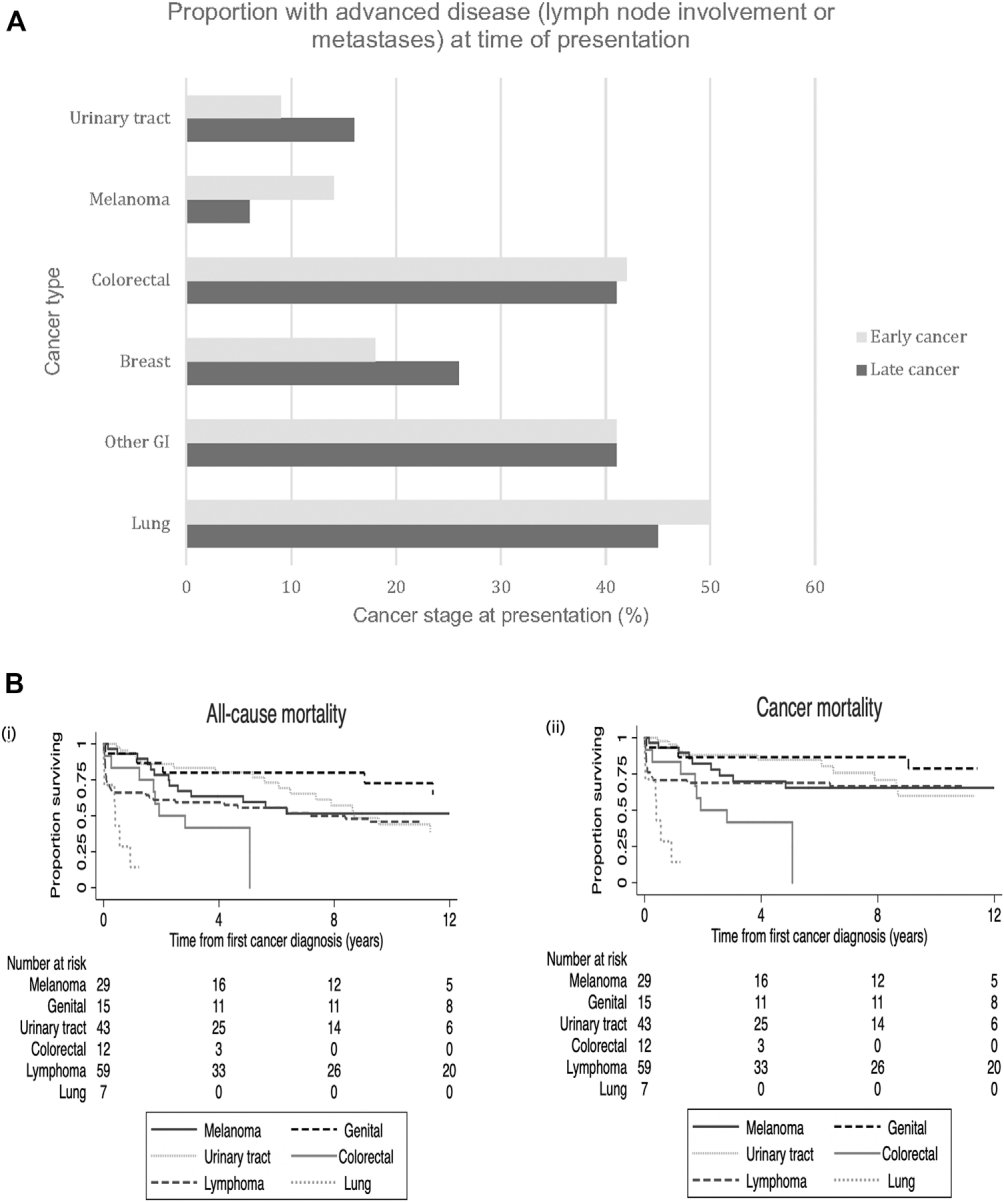
**(A)** Site-specific cancer types. Proportion of early site-specific cancers presenting with advanced stage disease (lymph node involvement or metastases) at time of cancer presentation in those with early vs. late *de novo* cancers. Legend: GI- gastrointestinal. **(B)** Kaplan Meier survival curves with number at risk tables for all-cause mortality (i) and cancer mortality (ii) according to the six common site-specific early *de novo* cancers.

Of recipients who had early-onset cancers, 25% (*n* = 61) developed or presented with advanced stage disease (lymph node involvement or distant metastases). At the time of presentation, 50% of lung, 42% of colorectal and 17% of breast cancers had evidence of advanced disease (Figure 2A). For late-onset cancers, 45% of lung, 41% of colorectal and 26% of breast cancers had evidence of advanced disease at the time of diagnosis. In contrast, 9% and 14% of early kidney cancers and melanomas and 16% and 6% of these late cancers respectively, presented with evidence of advanced disease. Kaplan-Meier survival curves for all-cause mortality and cancer mortality according to the most common site-specific cancer types are shown in Figure 2B.

**FIGURE 3:**
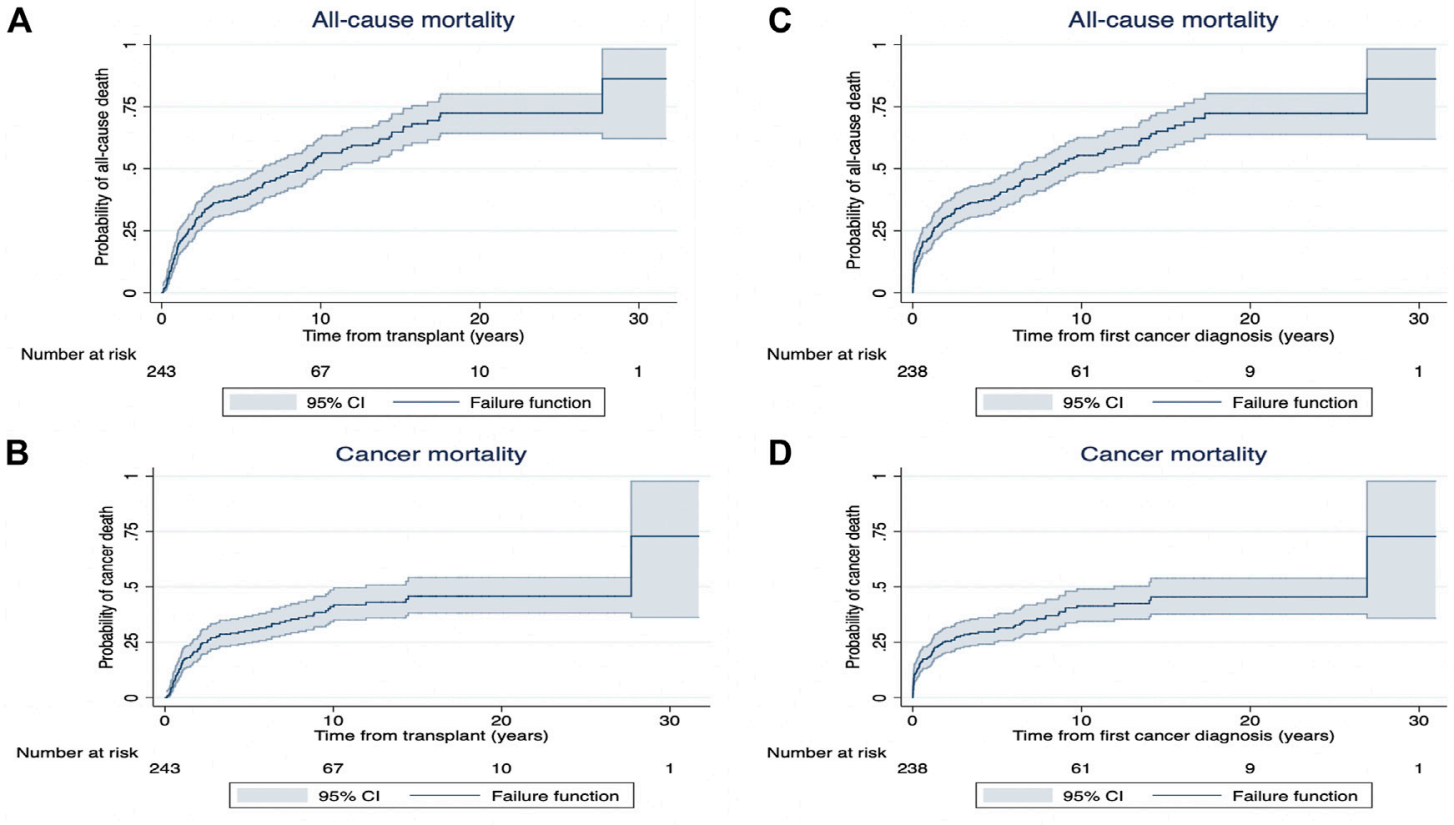
Kaplan Meier survival curves for all-cause mortality **(A)** and cancer mortality **(B)** from time of exposure (from transplant [years]) and all-cause mortality **(C)** and cancer mortality **(D)** from time from first cancer diagnosis (years) in recipients with early *de novo* cancer.

**FIGURE 4:**
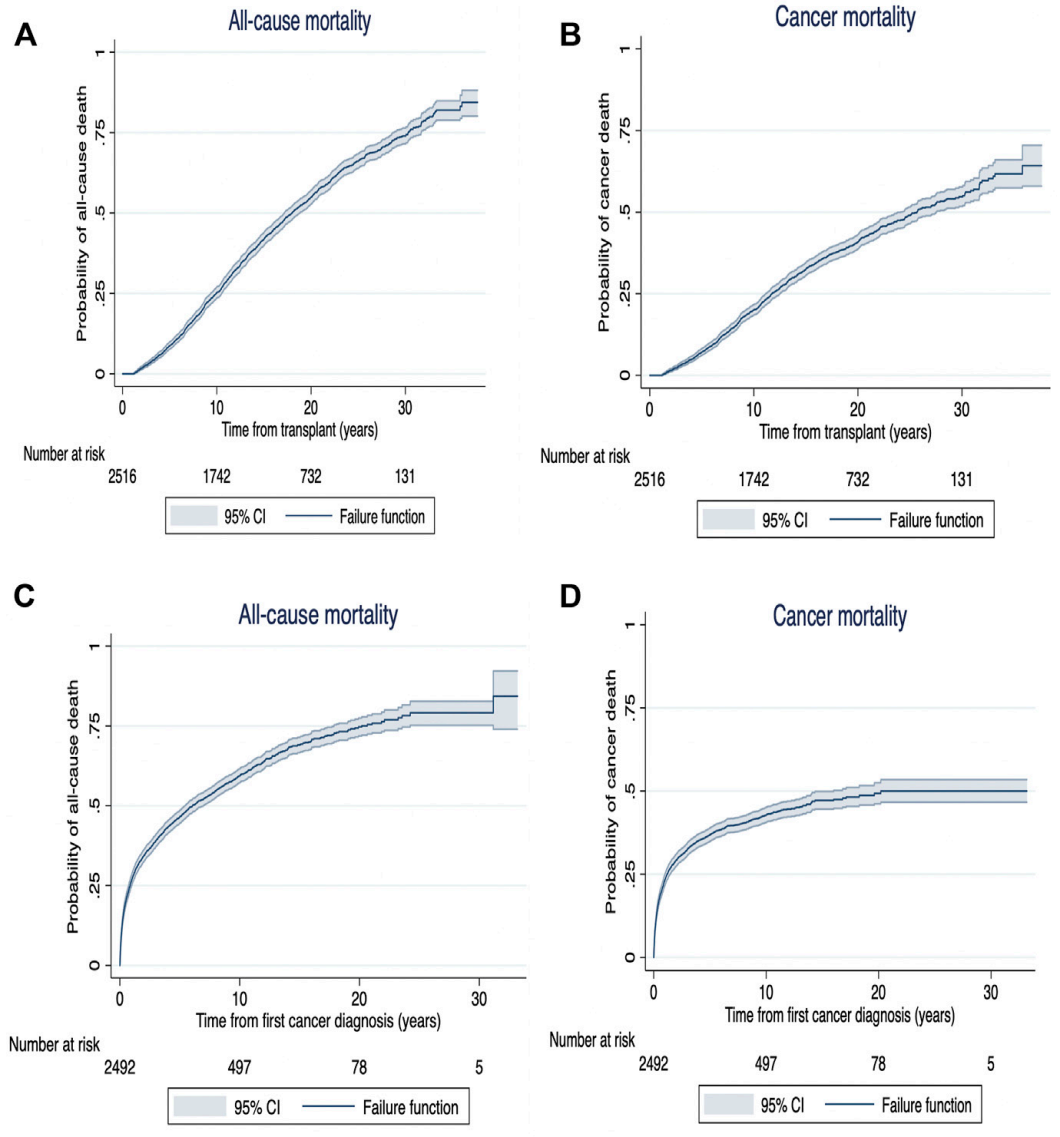
Kaplan Meier survival curves for all-cause mortality **(A)** and cancer mortality **(B)** from time of exposure (from transplant [years]) and all-cause mortality **(C)** and cancer mortality **(D)** from time from cancer diagnosis (years) in recipients with late (>12 months) *de novo* cancer.

Among recipients who developed *de novo* early-onset cancers, 77 (32%) died with 70 (91%) deaths attributed to cancer related deaths (Table 3). The median (IQR) time from cancer diagnosis to cancer-specific and all-cause deaths was 145 days (IQR: 20–464) and 144 days (20–505), respectively. For recipients with late-onset cancers, 1,473 (59%) recipients died with 977 (39%) attributed to cancer-related deaths. The median time from cancer diagnosis to cancer-specific and all-cause deaths were 229 days (53–781) and 427 days (80–1,623), respectively. Figures 3, 4 shows the Kaplan Meier curves of cancer-related deaths and all-cause deaths for recipients with early and late-onset *de novo* cancer; both from time of transplant and from time of cancer diagnosis, with majority of deaths being related to cancer in those who developed early *de novo* cancer.

**TABLE 3:** Outcomes of early cancer.

	Multiple incident cancers (*n*, %)	Number of cancers (*n*)	First cancer causing allograft failure (*n*, %)	First cancer causing Death (*n*, %)
Cancer first 12 months	39 (16.0)	243	3 (1.2)	77 (31.7)
Melanoma		29	0	7 (24.1)
Urinary tract^a^		44	1 (2.3)	7 (15.9)
Lymphoproliferative disease		62	1 (1.6)	25 (40.3)
Colorectal		12	0	7 (58.3)
Other GI^b^		17	0	14 (82.4)
Lung		8	0	6 (75.0)
Brain		3	0	1 (33.3)
Genital^c^		15	0	1 (6.7)
Prostate		9	0	1 (11.1)
Breast		12	0	0
Thyroid		7	0	0
Lip		1	0	0
Unknown origin		2	0	2 (100.0)
Others		22	1 (4.5)	6 (27.3)

Cancer occurring >12 months post-transplant: 13.4% multiple cancers, 1.2% first cancer causing allograft failure and 31.7% first cancer causing death.

a Cancers of the kidney, ureters and bladder.

b Other gastrointestinal tract cancers (including gall bladder, small intestine, bile duct, pancreas, liver, stomach, esophagus).

c Cervix, ovaries, uterus, penile.

Following cancer diagnosis, the overall patient survivals at 1, 5 and 10 years for recipients who developed early *de novo* cancer were 77% (95%CI: 70.7, 81.4), 46% (95%CI: 39.7, 52.2) and 25% (95%CI: 19.8, 30.7). In recipients who developed late *de novo* cancers, the overall patient survivals at 1, 5 and 10 years were 73% (95%CI: 70.7, 74.2), 41% (95%CI: 38.6, 42.3) and 20% (95%CI: 18.2, 21.3) (Supplementary Figure S1).

### Early-Onset Cancers Outcomes-Deaths, Recurrent and Second Cancers

Most of the early cancer related deaths were associated with lung (75%) and colorectal (58.3%) cancers (Table 3).

Of those who developed lung cancer, 75% (*n* = 6) died, with the median (IQR) time to death of 142 days (6–236). The median (IQR) age at diagnosis was 56 years (50–62) with 50% being males. 50% (*n* = 3) of the patients presented with advanced stage disease at the time of presentation.

Of those who developed early colorectal cancer, 58% (*n* = 7) died, with the median (IQR) time to death of 651 days (96–924). The median (IQR) age at diagnosis was 59 years (49–69) with 57% being males. 57% (*n* = 4) of the recipients presented with advanced stage disease at the time of presentation.

Of the more common cancer types, 25 (40%) recipients died from PTLD (median [IQR] age at diagnosis was 48 [28–56] years) while 7 (16%) died from urinary tract cancers (median [IQR] age at diagnosis was 56 [49–60] years), with nearly 50% of those dying from the latter presenting with advanced stage disease. A detailed description of all the early onset *de novo* cancers that had contributed to premature mortality is shown in Table 3.

Of all recipients with early-onset cancers (*n* = 243), onset of a second (new) cancer or recurrence of *de novo* cancer occurred in 39 recipients (16%), with a median (IQR) time to cancer occurrence of 1,165 (71–2,309) days. The most common cancers were those that involved the urinary tract, lung and the gastrointestinal tracts (15% each). Seven (18%) of these second cancers occurred within 1 year of the primary malignancy.

In this cohort, 77% (*n* = 33) recipients developed a second new malignancy at a different site within a median (IQR) of 1,414 (435–2,423) days, of which, the most common cancer sites were lung cancer (*n* = 6, 18%) closely followed by cancer of the urinary tract (*n* = 5, 15%). Additionally, 15% (*n* = 6) recipients had recurrence of the *de novo* primary malignancy within a median (IQR) of 5 (0–159) days, of which 33% (*n* = 2) had recurrence of melanomas and 33% (*n* = 2) had recurrence of transitional cell cancer of the urinary tract.

Treatments of cancers that resulted in death were diverse and included various combinations of surgical resection, chemotherapy, radiotherapy and reduction of immunosuppressive medications (Table 4).

**TABLE 4:** Characteristics of early cancers contributing to death.

Cancer type, Sex distribution and median age (IQR)	Initial cancer location, type and number	Stage at presentation/number/time to advanced disease	Treatment
Melanoma (*n* = 7), M3, F4, Median age 61 (54–62)	Skin-7	Metastatic lesion- 6 (2 metastasized at initial presentation	Local excision- 6
		- 4 metastasized later; Median 15 months [12–27])	Chemotherapy and reduction IS- 1
			No treatment- 1
Urinary tract (*n* = 7)^a^, M6, F1, Median age 56 (49–60)	Urinary bladder- 5 (3x TCC, 2x adenocarcinoma)—4 invasive presentation)	Metastatic lesions- 3	Bladder excision-2 Chemotherapy- 1
	Native kidney-2 (adenocarcinoma)	- Kidney: 1- at presentation	Kidney mass excision- 1
		- Bladder:2- at 7 and 10 months	
Lymphoproliferative disease (*n* = 25), M16, F9, Median age 48 (28–56)	Lymph nodes/blood/bone marrow- 10	*In situ* lesion- 4	Reduced IS- 10
	Liver- 2	Invasive lesion- 7	Chemotherapy- 5
	Small intestine- 2	Metastatic lesion- 12 (all at presentation)	Radiotherapy- 5
	Brain- 3	Local lymph nodes- 1	Local excision (small intestine- 1)
	Colon- 2	Unknown- 1	No treatment- 8
	Kidney- 2		Unknown- 1
	Lung- 2		
	Unknown primary- 2		
Colorectal (*n* = 7), M4, F3, Median age: 59 (49–69)	Colon- 5	Invasive- 3	Local excision- 3
	Recto-sigmoid- 2 (adenocarcinoma- 1, squamous cell carcinoma- 1)	Metastatic lesion- 4 at presentation	Radiotherapy-1
		All 3 invasive metastasized at 8, 13 and 55m post-diagnosis	Chemotherapy- 3
			No treatment- 1
Other GI (*n* = 14)^b^, M 11, F3, Median age: 59 (35–66)	Pancreas- 5 (adenocarcinoma)	Metastatic lesions- 8 (6 at presentation: pancreas- 3, oesophagus- 2, small bowel- 1, stomach- 1)	Pancreas: none- 2, reduction in IS- 2, chemotherapy- 1
	Stomach- 2 (adenocarcinoma)	Invasive- 4	Stomach: none- 1, chemotherapy- 1
	Oesophageal- 2, (adenocarcinoma)		Oesophageal: Chemo-radiotherapy- 2
	Ampulla of Vater- 2		Ampulla of Vater: None- 2
	Hepatocellular carcinoma- 1		Small intestine: Excision- 1
	Small intestine- 1 (adenocarcinoma)		Oropharynx: Excision, reduction IS and radiotherapy- 1
	Oropharynx- 1		HCC: excision- 1
Lung (*n* = 6), M3, F3, Median age: 56 (50–62)	Adenocarcinoma- 2	Metastatic lesion- 4 (3 at presentation, 1 at 4 months)	Radiotherapy- 2
	Small cell cancer- 2 Large cell cancer- 1	Invasive- 2	Chemotherapy- 1
	Unknown- 1		Multiple- 2 (chemo-radiotherapy and reduction IS- 1)
			None- 1

M, males, F, females.

a Cancers of the kidney, ureters and bladder

b Other gastrointestinal tract cancers (including gall bladder, small intestine, bile duct, pancreas, liver, stomach, esophagus).

### Factors Associated With All-Cause Mortality in Early-Onset Cancers

Risk factors associated with all-cause death among those with early cancers were older recipient age [>55 years: 2.42 (1.49–3.94), ref: 35-55 years] and older donor age [1.18 (1.03–1.36), per 10-years].

## Discussion

In this large contemporaneous cohort of kidney transplant recipients with *de novo* cancers spanning over 3 decades, we have shown that almost 1 in 10 of these cancers occurred within the first 12 months post-transplantation. The most common cancer types were PTLD, malignant melanoma and cancers of the urinary tract, and typically, most of these cancers were of early stage at the time of presentation. On the contrary, recipients with other cancer types such as cancers of the digestive and respiratory systems tend to present with advanced stage disease. Overall, 32% of patients with early cancers died within a median of 4.7 months (IQR: 0.6–16) post-diagnosis and 91% were cancer-related deaths. Characteristics associated with an increased risk of death in recipients with early-onset cancer included increasing donor and recipient age.

Early cancers after transplantation are devastating events with a high burden of morbidity and mortality. Additionally, treatment strategies lack robust trial-based evidence and usually consist of surgical resection, radiotherapy and judicious reduction in immunosuppression with regular monitoring for cancer progression and allograft function. Certain strategies such as cancer screening may reduce the incidence of late-stage cancer through early detection, allowing interventions to be instigated early and before transplantation when the disease is still at a precancerous stage. Most clinical practice guidelines recommended routine age and sex-specific population-based cancer screening prior to listing (7). These include biennial bowel screening using either fecal immunochemical testing, or 5-years flexible sigmoidoscopy, biennial mammography for breast cancer, low-dose computer-tomography for lung cancer screening, and routine cervical screening using human papillomavirus test (HPV) for oncogenic cervical genotypes and pre-cancerous cervical lesions prior to transplantation (7). Despite these recommendations, uptake for screening in general among our candidates with chronic kidney disease is likely to be low and may potentially explain the late presentation of certain cancer types such as lung and gastrointestinal cancers within the early months after transplantation. While we do not routinely collect screening data in our transplant candidates, our prior work has indicated that the uptake of certain cancer screenings such as breast and cervical cancer are quite low amongst patients with kidney disease (11). Patients with kidney disease and kidney transplants undergo significant changes to their overall physical and psycho-social health and tend to focus on their current kidney health and are less inclined to prioritize cancer screening over imminent health problems.

Other guidelines suggest routine ultrasonographic screening (either annual or biennially) for renal cell cancers. However, evidence to support these recommendations are limited. For instance, the accuracy of ultrasonography in detecting malignant lesions in those with kidney failure is uncertain. Ultrasonography is largely operator-dependent and test performance varies with patient habitus, the kidneys and the size of the lesion (12). In the general population, test sensitivity and specificity are lower in detecting tumours <3 cm in size. In patients with kidney failure, who have scarred native kidneys with acquired cystic disease, the accuracy of detecting small renal cell cancers is ambiguous. Moreover, prior Markov modelling studies have suggested that routine surveillance for renal cancers may not be cost effective in the low to moderate risk population (13). Screening is not without harm as uncertain lesions may lead to further investigations or treatments, and therefore undue delays for transplant waitlisting. Currently, there is no clear consensus on screening for post-transplant renal cell cancers as data are limited (14). There are similar concerns regarding the risk-benefit ratio of screening high risk population for lung cancers with annual low-dose computed tomography even in the general population (15) and this modality has not been validated in the transplant population.

Viral linked cancers such as lymphoma or post-transplant lymphoproliferative disorders (PTLD) have a higher incidence in the transplant population, compared to the general population, with standardized mortality rates (SMRs) being as high as 10.7 for PTLD (3, 6, 16). A quarter of our cohort with early cancers developed PTLD within the first year of transplant with a younger median age of 48 years compared to other cancers and nearly 40% died. This is consistent with previous findings of a bimodal distribution in the incidence of PTLD development after transplantation (17). PTLD most commonly occurs within the first year of transplant affecting younger (<25 years) or older (>60 years) patients (18) and has a high mortality rate of ∼50% (19, 20). Primary Epstein-Barr virus (EBV) infection and pre-transplant EBV sero-negativity are risk factors for early onset PTLD, especially in younger transplant recipients, while late B-cell PTLD involves EBV-negative lesions (in 40–50%) (17). Once PTLD occurs, the risk of death is high (>14 fold higher than in recipients without PTLD) with median time of 6 months from diagnosis to death (21).

Older recipient age and donor age were both associated with an increased risk of cancer-related death. Over the past decades, there has been a changing demographic of transplant recipients. We are increasingly transplanting older patients with higher comorbidity burden and this in turn may have implications on screening procedures, cancer monitoring and degree of immunosuppression.

This study has several limitations. ANZDATA registry does not collect information on the uptake, adherence, type and timing of cancer screening for each transplant recipient. We lacked information on histological cancer data and treatment specific data, EBV data, relevant habits such as tobacco or alcohol use, therapeutic drug levels of immunosuppressive drugs, patients who were listed for kidney transplantation or were subsequently delisted (including those who may have developed incident cancer on the waiting list), quality of life measures and the severity of comorbid disease. There is a likelihood of selection bias due to systematic differences in the management of recipients who developed cancer.

## Conclusion

In conclusion, early cancer is an infrequent complication after kidney transplantation but once it occurs, outcomes are generally poor. Clinicians should be more cognizant of the development of early cancers especially in the older population. Examination of granular data and the development of screening and management approaches to decrease post-transplant cancers without increasing the risk of allograft failure, with clear considerations of patient preferences and values may improve outcomes in this population.

## Data Availability

The data analyzed in this study is subject to the following licenses/restrictions: Data was obtained from the Australian and New Zealand Dialysis and Transplant registry. Requests to access these datasets should be directed to https://www.anzdata.org.au.

## Supplementary Material

Supplementary Figure S1 Proportion of recipients who developed a *de novo* cancer post-transplant stratified by transplant era.

## Abbreviations

ANZDATA, Australian and New Zealand dialysis and transplant; ANOVA, analysis of variance; EBV, epstein-barr virus; ESKD, end stage kidney disease; HLA, human leukocyte antigen; ICD, international classification of disease; IQR, inter-quartile range; NMSC, non-melanoma skin cancer; PTLD, post-transplant lymphoproliferative disease; SD, standard deviation; SMR, standardized mortality rates.
