# Supplementary figures and images for: Incidence and Outcomes of Early Cancers After Kidney Transplantation
Source: Transpl Int. 2022 May 3;35:10024. doi: 10.3389/ti.2022.10024 (PMC9110645; doi:10.3389/ti.2022.10024)

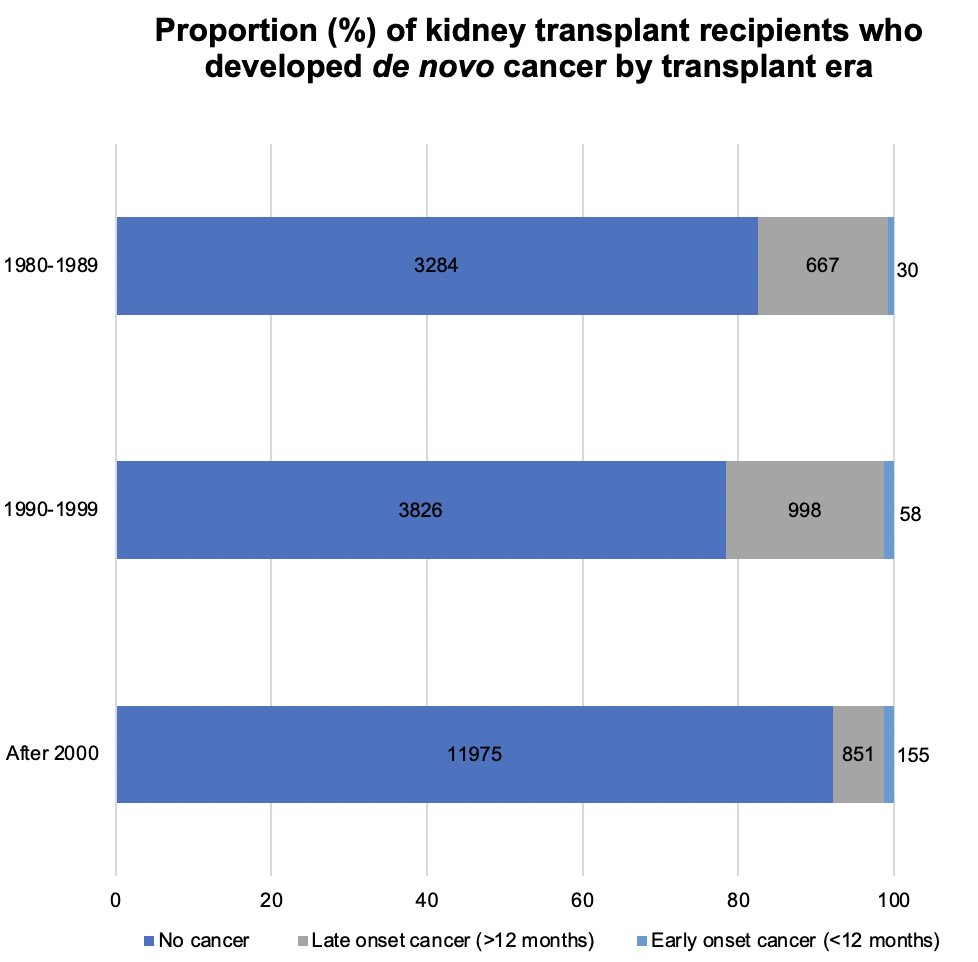

Supplement: Supplementary file 1 [file Image1.TIFF]
