# Supplementary material for: Incidence and Outcomes of Early Cancers After Kidney Transplantation
Source: Transpl Int. 2022 May 3;35:10024. doi: 10.3389/ti.2022.10024 (PMC9110645; doi:10.3389/ti.2022.10024)
Supplement: Supplementary file 2 [file DataSheet1.docx]

**Table S1. Characteristics of recipients who developed early *de novo* cancer by transplant era**

| Characteristics (n,%) | Transplant Era | | | |
| --- | --- | --- | --- | --- |
|  | **1980-1989 (n=30)** | **1990-1999 (n=58)** | **After 2000 (n=155)** | **p-value** |
| Recipient age, years (mean, SD)  Gender  Female  Male  Presence of comorbidities  Diabetes  Cerebrovascular disease  Coronary artery disease  Peripheral vascular disease  Wait Time  Under 4 years  4 years or more | 49.5 (9.4)  16 (53)  14 (47)  2 (7)  1 (3.3)  1 (3.3)  0 (0)  27 (90)  3 (10) | 45.3 (18.6)  33 (57)  25 (43)  5 (8.6)  3 (5.2)  3 (5.2)  3 (5.2)  51 (88)  7(12) | 53.1 (14.6)  49 (32)  106 (68)  33 (22)  5 ((3.2)  26 (17)  7 (4.5)  122 (79)  33 (21) | <0.01  0.001  0.17  0.21  0.22  0.78  0.15 |

**Table S2: Characteristics of recipients and types of early *de novo* cancers by type of transplant**

| Characteristics | Living n=64 | Deceased n=179 | p value |
| --- | --- | --- | --- |
| Age at transplant (mean, SD)  Waiting time (years, mean, SD)  Types of cancer^1^ (n,%)  Lymphoproliferative disease  Urinary tract cancer  Melanoma  Other GI tract  Colorectal  Lung | 45.2 (21)  1.3 (1.7)    20 (31)  10 (16)  11 (17)  3 (5)  6 (9)  1 (2) | 52.8 (12.3)  2.8 (2.1)  42 (23)  34 (19)  18 (10)  14 (8)  6 (3)  7 (4) | <0.001  <0.001  0.21  0.59  0.13  0.43  0.05  0.45 |

1= Most common cancer types
